# An external quality assessment feasibility study; cross laboratory comparison of haemagglutination inhibition assay and microneutralization assay performance for seasonal influenza serology testing: A FLUCOP study

**DOI:** 10.3389/fimmu.2023.1129765

**Published:** 2023-02-28

**Authors:** Joanna Waldock, Carol D. Weiss, Wei Wang, Min Z. Levine, Stacie N. Jefferson, Sammy Ho, Katja Hoschler, Brandon Z. Londt, Elisa Masat, Louise Carolan, Stephany Sánchez-Ovando, Annette Fox, Shinji Watanabe, Miki Akimoto, Aya Sato, Noriko Kishida, Amelia Buys, Lorens Maake, Cardia Fourie, Catherine Caillet, Sandrine Raynaud, Richard J. Webby, Jennifer DeBeauchamp, Rebecca J. Cox, Sarah L. Lartey, Claudia M. Trombetta, Serena Marchi, Emanuele Montomoli, Iván Sanz-Muñoz, José María Eiros, Javier Sánchez-Martínez, Danny Duijsings, Othmar G. Engelhardt

**Affiliations:** ^1^Vaccines, Science Research & Innovation, Medicines and Healthcare Products Regulatory, Potters Bar, United Kingdom; ^2^Center for Biologics Evaluation and Research, US Food and Drug Administration, Silver Spring, MD, United States; ^3^Influenza Division, Centers for Disease Control and Prevention, National Center for Immunization and Respiratory Diseases, Atlanta, GA, United States; ^4^Respiratory Viruses Unit, UK Health Security Agency, Colindale, United Kingdom; ^5^hVivo The Queen Mary Bioenterprises (QMB) Innovation, London, United Kingdom; ^6^World Health Organisation (WHO) Collaborating Centre for Reference and Research on Influenza, Royal Melbourne Hospital, at the Peter Doherty Institute for Infection and Immunity, Melbourne, VIC, Australia; ^7^Department of Infectious Diseases, University of Melbourne at the Peter Doherty Institute for Infection and Immunity, Melbourne, VIC, Australia; ^8^Center for Influenza and Respiratory Virus Research, National Institute of Infectious Diseases: Musashi-Murayama, Tokyo, Japan; ^9^Centre for Respiratory Diseases and Meningitis, National Institute for Communicable Diseases (NICD) of the National Health Laboratory Services, Johannesburg, South Africa; ^10^Department of Research and Development, Sanofi, Lyon, France; ^11^Department of Infectious Diseases, St Jude Children’s Research Hospital, Memphis, TN, United States; ^12^Influenza Centre, Department of Clinical Sciences, University of Bergen, Bergen, Norway; ^13^Department of Molecular and Developmental Medicine, University of Siena, Siena, Italy; ^14^National Influenza Centre of Valladolid, Instituto de Estudios de Ciencias de la Salud de Castilla y León (ICSCYL), Edificio Rondilla, Hospital Clínico Universitario de Valladolid, Valladolid, Spain; ^15^Viroclinics, Clinical Virology Services, Rotterdam, Netherlands

**Keywords:** influenza viruses, external quality assessment (EQA), haemagglutination inhibition (HAI), microneutralization (MN), serology, standardization

## Abstract

**Introduction:**

External Quality Assessment (EQA) schemes are designed to provide a snapshot of laboratory proficiency, identifying issues and providing feedback to improve laboratory performance and inter-laboratory agreement in testing. Currently there are no international EQA schemes for seasonal influenza serology testing. Here we present a feasibility study for conducting an EQA scheme for influenza serology methods.

**Methods:**

We invited participant laboratories from industry, contract research organizations (CROs), academia and public health institutions who regularly conduct hemagglutination inhibition (HAI) and microneutralization (MN) assays and have an interest in serology standardization. In total 16 laboratories returned data including 19 data sets for HAI assays and 9 data sets for MN assays.

**Results:**

Within run analysis demonstrated good laboratory performance for HAI, with intrinsically higher levels of intra-assay variation for MN assays. Between run analysis showed laboratory and strain specific issues, particularly with B strains for HAI, whilst MN testing was consistently good across labs and strains. Inter-laboratory variability was higher for MN assays than HAI, however both assays showed a significant reduction in inter-laboratory variation when a human sera pool is used as a standard for normalization.

**Discussion:**

This study has received positive feedback from participants, highlighting the benefit such an EQA scheme would have on improving laboratory performance, reducing inter laboratory variation and raising awareness of both harmonized protocol use and the benefit of biological standards for seasonal influenza serology testing.

## 1 Introduction

External Quality Assessment (EQA) schemes are an important tool for evaluating inter-laboratory agreement in testing biological assays. They allow for comparison of a laboratory’s performance with a source outside of that laboratory – in the case of this study, with a group of peer laboratories. This provides objective evidence of a laboratory’s performance and can be used to identify where greater standardization and/or method improvements are required. Alongside the use of standardized protocols and the provision of biological standards, EQAs indirectly help to reduce inter-laboratory variability. There are currently no international EQA schemes for influenza serology testing as far as the authors of this paper are aware. The ECDC/WHO run a European External Influenza Virus Quality Assessment Programme, which assesses virus isolation, antigenic and genetic characterization methods (1). In the ECDC/WHO EQA serology methods are used to type and identify unknown strains of influenza. This assesses the qualitative results of Haemagglutination Inhibition (HAI) testing using monospecific ferret sera and is aimed at virus identification. However testing for quantifying antibodies in human sera is not assessed. When using serology assays to define antibody correlates of protection, accurate and quantitative results are of the highest importance.

FLUCOP (http://www.FLUCOP.eu/) is a joint European project between academia, vaccine manufacturers and public health authorities, supported by the Innovative Medicines Initiative Joint Undertaking (IMIJU) aimed at standardizing serological assays and developing harmonized protocols for evaluating influenza vaccines. The goal of the FLUCOP project is to have a direct and evidence-based impact on the quality of regulatory decisions and to provide valid and appropriate serological tools for the future definition of alternative correlates of protection for (novel) influenza vaccines. The consortium has made considerable progress with standardization of the HAI assay and Enzyme-Linked-Lectin Assay (ELLA) with large collaborative studies carried out and freely available published SOPs (2, 3). Additionally collaborative studies to standardize the Microneutralization (MN) assay have also been carried out (manuscript in preparation).

Alongside large studies testing harmonized serology assay protocols and the impact of biological standards on inter-lab variability, the FLUCOP consortium set up an EQA feasibility study. In this study we assessed two common assays used for influenza serology: the HAI and MN assays. Participants were asked to test a provided panel of 30 serum samples using four seasonal influenza viruses. This study aimed to determine interest in EQA schemes for influenza serology testing, provide a snapshot of inter-laboratory variation outside the limits of the FLUCOP consortium, assess the use of a serum standard to reduce inter-laboratory variation and provide useful feedback to participants for identifying issues and improving proficiency.

## 2 Materials and methods

### 2.1 Participating laboratories

42 Laboratories were invited to participate in the EQA feasibility study. Invited laboratories were from industry, contract research organizations (CROs), academia and public health institutions who regularly conduct HAI and MN testing and have an interest in serology standardization. We received a positive response from 17 participants (a number likely negatively impacted by the COVID-19 pandemic).

### 2.2 Serum panels and serum standards

Participants were provided with a panel of 30 samples by the University of Ghent. Each panel consisted of 3 pre and 20 post-vaccination human sera (FLUCOP_QIV clinical trial, Fluarix Tetra vaccine containing the following influenza strains: A/Michigan/45/2015 (H1N1)pdm09, A/Hong Kong/4801/2014 (H3N2), B/Brisbane/60/2008 and B/Phuket/3073/2013 samples), 4 duplicated samples (for assessing within-run variability), 2 serum standards and a negative control. The two serum standards included in the study were pools of equal volumes of 4 post-vaccination human serum samples (FLUCOP_QIV clinical trial, Fluarix Tetra vaccine) all of which have med-high titres for all tested influenza A subtype and B lineage viruses. These serum standards were used as calibrators to normalise data from each laboratory (i.e. normalised titres from each testing laboratory are expressed relative to the titre of the serum standard, see statistical analysis). Prior to this study serum samples were pre-screened in HAI/MN and selected to cover a large range in titres. All sera were heat inactivated at 56^°^C for 1 hour. An IgA/IgM/IgG depleted human sera was used as a negative control (Sigma-Aldrich S5393).

### 2.3 Serological testing

Participants were asked to test the serum panel with four seasonal influenza viruses. They were asked where possible to use reassortant viruses IVR-180 (H1N1), NYMC-X263B (H3N2), NYMC-BX35 (B Victoria lineage) and NYMC-BX59A (B Yamagata lineage). Antigenically identical wild type (WT) viruses were also considered acceptable for testing. Participants were asked to carry out three independent replicates using any or all of the following: In-house or FLUCOP protocols for HAI testing (2); In-house or the WHO protocol for 2-day ELISA-based MN testing (4); In-house or FLUCOP protocols for 3-day+ MN testing (available upon request). Serum panels were shipped in August/September 2021. Each laboratory returning data was assigned a number (and a colour for all graphical representations of data); each laboratory was only given their own number and colour, thus anonymizing the results from participating laboratories.

### 2.4 Statistical analysis

As HAI and some MN methods return discrete data within a specific dynamic range, results are often reported as <10 or >(upper assay limit) e.g.>1280. Data returned indicating <t were assigned the value 1/2t, data returned indicating >t were assigned the value 2*t. Intra-assay (within-run) variability was assessed using maximum-minimum ratios of four coded duplicate samples included in the serum panel. Intra-laboratory (between-run) variation was assessed using maximum-minimum ratios of the 3 independent replicates returned for HAI (with the exception of Lab no.13 where only 2 replicate runs were returned) or 2-3 independent replicates for MN (Labs no.7/10/11 returned two replicate runs). Any sample with a ratio greater than 3.5 was excluded from inter-laboratory (between-laboratories) comparisons. Any sample with a Geometric Mean Titre (GMT) <10 was excluded from statistical analysis (shown on graphs for information only). Sample titres were log_10_ transformed and % Geometric Coefficient of Variation (GCV) calculated using the following equation: (10^s^-1)x100% where s is the standard deviation of the log_10_ titres. %GCVs were statistically compared using the Wilcoxon matched paired t test (comparison of raw and normalized titres) or Mann-Whitney U test (comparison of native and ether split B antigen titres).

Geometric Mean Ratios (GMRs) were calculated as the titre of a sample/a reference titre. For overall inter-laboratory variability the reference titre is the GMT of that sample across all testing laboratories (after data exclusion as described above). For comparisons of in-house and FLUCOP protocols, the reference titre is the GMT of that sample tested with the appropriate protocol (i.e. the GMT of a sample across all laboratories testing with the FLUCOP protocol, or the GMT of a sample across all laboratories testing with in-house protocols).

For normalization of titres, from the two serum standards (pools of post-vaccination serum as previously described) included in the panel, we selected the serum standard for which the greatest number of valid data were returned - sample no.17. For each run a calibration factor was calculated as the ratio of titre of sample no.17 in a run/the global GMT of sample no.17 (GMT across all testing runs/laboratories after data exclusion as described above). The calibration factor was then applied to all other titres within that run to calculate normalized titres.

Each participant received a report giving an overview of all returned data, with laboratory specific information for the following: intra-assay variability (maximum-minimum duplicate sample ratios %>3.5 by subtype/lineage compared to the average across testing laboratories), intra-laboratory variability (maximum-minimum ratios of sample titres across runs %>3.5 by subtype/lineage compared to the average across testing laboratories) and inter-laboratory variability (GMRs pre- and post- normalization by subtype/lineage compared to the average across testing laboratories).

## 3 Results

### 3.1 Returned data

Of the 17 accepted participants 16 laboratories returned data by the study deadline. One participant dropped out of the study due to administrative issues. For HAI analysis 13 laboratories returned data. One laboratory was excluded as only a single replicate was carried out. Six laboratories carried out both FLUCOP and in-house HAI testing, giving 19 data sets in total. One laboratory carried out two runs (of both FLUCOP and in-house testing). Where two-three independent replicates were carried out, data was taken forward for analysis. For MN testing 8 laboratories returned data. Four laboratories carried out in-house testing, two laboratories used the WHO ELISA-based MN assay (hereafter designated FLU in figures) and two laboratories carried out both in-house and the WHO ELISA-based MN assays, giving 10 data sets in total. It should be noted that laboratory 4 returned MN data for the B lineage viruses only, and laboratory 5 returned in-house MN data for the H3N2 influenza A virus only.

### 3.2 HAI testing results

#### 3.2.1 Intra-assay variability

We conducted an intra-assay (or within run) variability analysis by comparing the maximum to minimum ratio of 4 pairs of coded duplicates within the serum panel. Duplicate ratios of equal to or less than 2 (≤2) were considered acceptable. In the data returned over 98% of duplicate ratios were ≤2 (73% of the ratios were =1 i.e. the duplicate samples had the same value). Intra-assay performance was good across all laboratories (with a small number of laboratories having higher incidence of duplicate ratios greater than 2)

#### 3.2.2 Intra-laboratory variability

**Figure 1:**
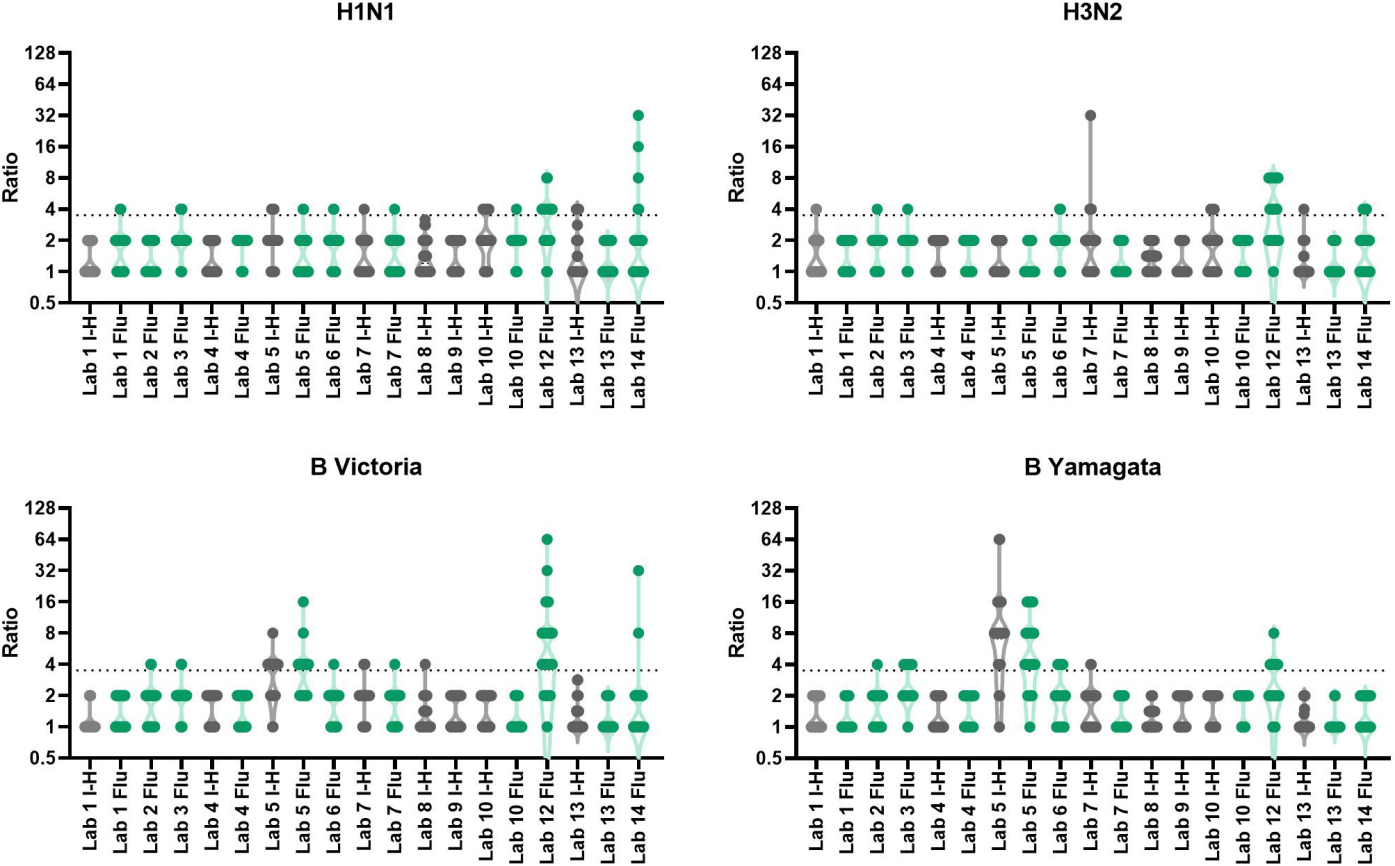
Intra-laboratory variability in HAI testing. Maximum to minimum titre ratios for three independent HAI replicates per laboratory (with the exception of Lab no.13 where only 2 replicate runs were returned). Dashed line represents the 3.5 cut-off for data exclusion for inter-laboratory variation analysis. Data in grey indicates in-house assays, data in green indicates FLUCOP assays.

We conducted an intra-laboratory (or between-run) variability analysis by comparing maximum to minimum ratios of all samples tested across the two-three independent replicates performed by the testing laboratories. **Table 1** shows the % of values falling within ratios of 1, 2, or ≥4. **Figure 1** plots the ratios per laboratory for each influenza subtype/lineage tested. Ratios were generally good for influenza A H1N1 and H3N2 strains, with more than 90% of ratios being <3.5. There is greater intra-laboratory variability for the B-strains, with some individual laboratories frequently obtaining ratios higher than 4. Samples with maximum to minimum ratios greater than 3.5 were excluded from further analysis (see statistical analysis). Overall, 11% of returned data was excluded, with the highest failure rate for B Yamagata (% excluded data H1N1: 9.5%, H3N2: 7.2%, B Victoria: 12.6% and B Yamagata: 15.1%).

**Table 1:** HAI Intra-laboratory variation - % of maximum to minimum titre ratios across at least 2 replicates that =1, = 2, or ≥4.

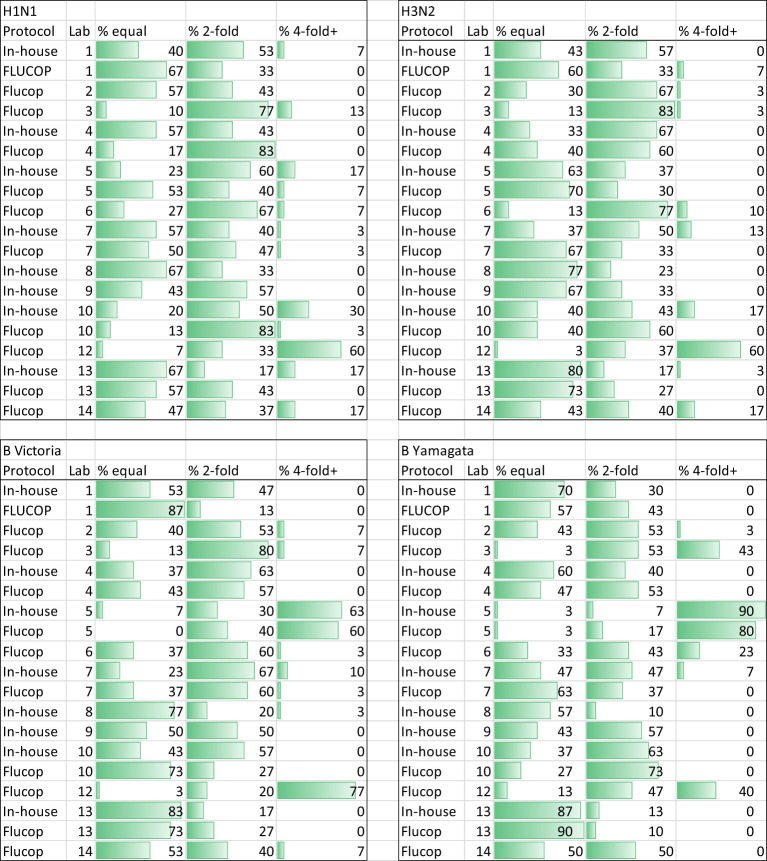

#### 3.2.3 Inter-laboratory variability

**Figure 2:**
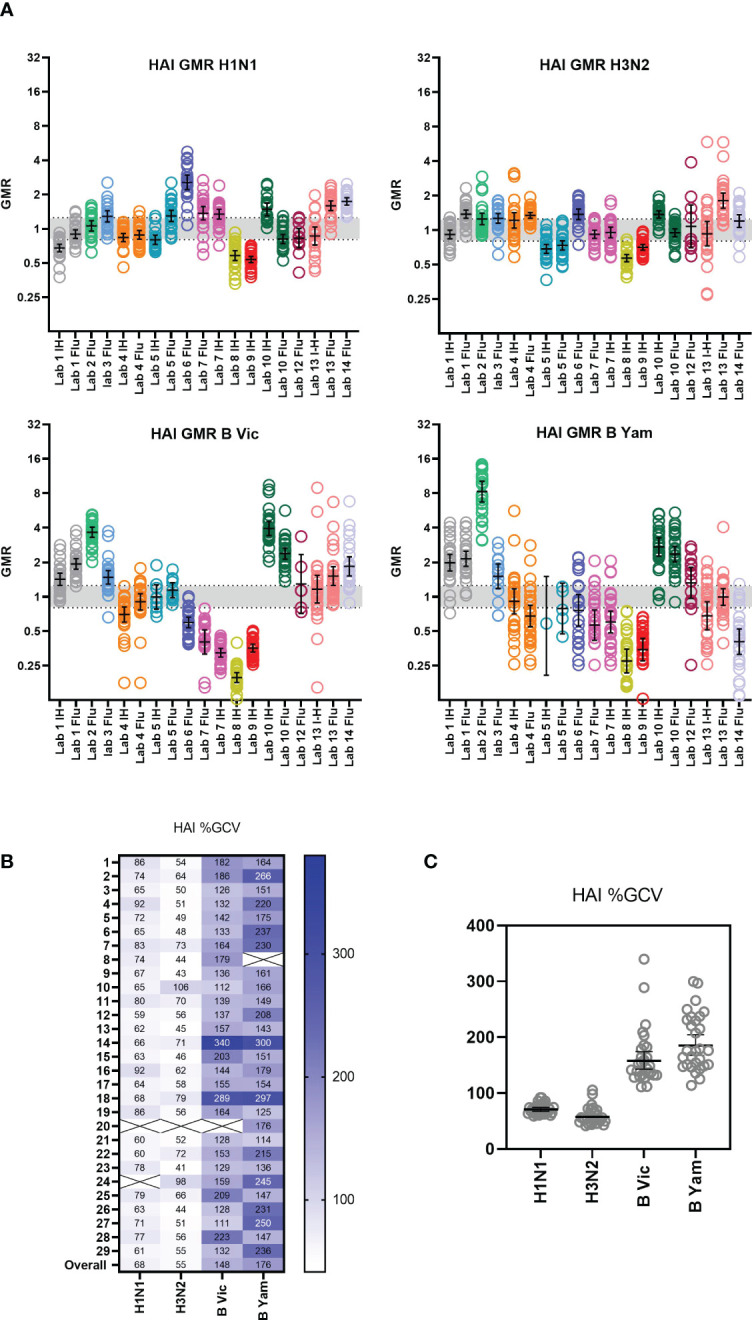
Inter-laboratory variation in HAI testing. **(A)** GMR of each sample HAI titre compared to the global GMT of that sample across all testing laboratories. Geometric mean and 95% CI are shown in black. The indicative interval of 0.8-1.25 is shaded in grey. Each laboratory has a unique number and colour in all graphical representation of data. **(B, C)**. %GCV of each sample HAI titre across the testing laboratories. Samples with a GMT <10 were excluded from analysis (shown as Xs in **B**).

An inter-laboratory (or between-laboratory) variability analysis was carried out by comparison of %GCV for each sample tested across all laboratories and a comparison of the GMR of each sample. GMRs were calculated as the ratio of the titre of a sample in a given laboratory/run divided by the global GMT of that sample across all testing laboratories. This gives a relative measure of agreement between the laboratories, where in a perfect world all titres for a sample are the same and thus all ratios are 1. Where a laboratory returns a titre twice the value of the global GMT, the ratio is 2, and conversely where a laboratory returns a titre half that of the global GMT, the ratio is 0.5. The indicative interval of 0.8-1.25 is considered to be acceptable, however this is an arbitrary range. **Supplementary Figure S1A** shows the overall returned HAI data (after data exclusion as described in statistical analysis) from all laboratories for each influenza A subtype and B lineage. Data from in-house testing is shown in black, data from FLUCOP testing is shown in red. **Figure 2** plots the GMR of each sample by laboratory (**Figure 2A**) and shows a heatmap of %GCV for each sample (**Figures 2B, C**).

Inter-laboratory variation was overall quite low for H1N1 and H3N2 testing, with overall %GCVs of 68 and 55 respectively. Both B Victoria and B Yamagata lineages show much higher inter-laboratory variation, with overall %GCVs of 148 and 176, respectively.

#### 3.2.4 Impact of a study standard on HAI inter-laboratory variation

We included two pools of human sera as study standards within the serum panel tested by participating laboratories. The impact of normalisation using these study standards was explored by comparison of %GCV of log_10_ titres before and after normalisation with the study standard and a comparison of GMRs before and after normalisation. Pool 1 was selected as more laboratories returned a valid titre for Pool 1 than Pool 2.

**Figure 3:**
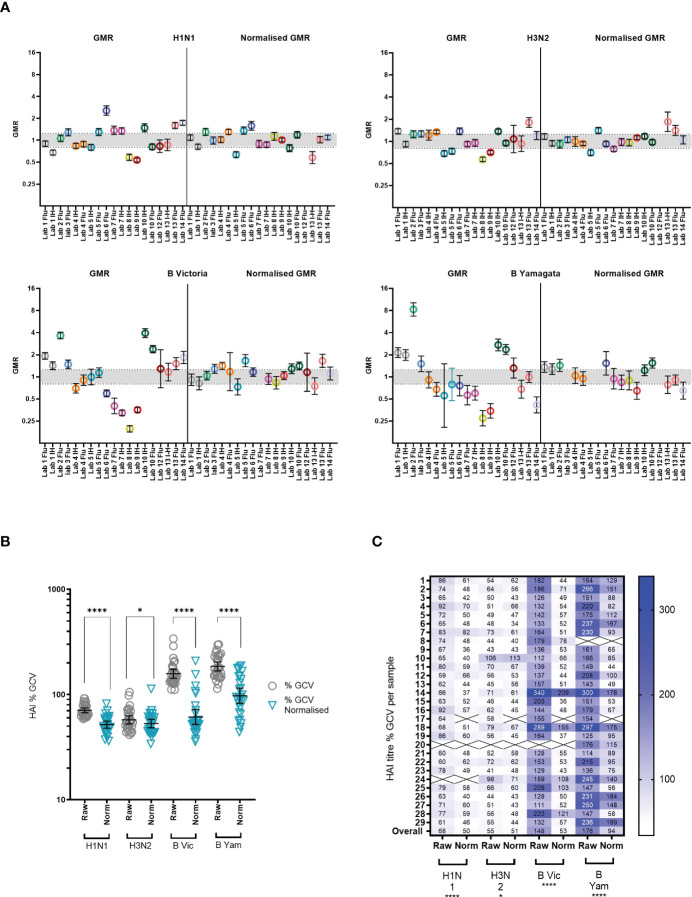
Impact of normalisation on inter-laboratory agreement in HAI testing. **(A)** GMR of HAI titres for each sample before and after normalisation using Pool 1 (sample 17) as a study standard. Negative samples (HAI GMT <10) were excluded from analysis. Where a laboratory did not return a valid titre for the study standard in each reported run, that laboratory was excluded from analysis (Lab no.12 (H1N1), Lab no.12 (H3N2), Lab no.7 (B Victoria) and Labs no.3 and 5 (B Yamagata)) Geometric mean (circles) and 95% CI (black lines) are shown. The indicative interval of 0.8-1.25 is shaded in grey. Each laboratory has a unique number and colour in all graphical representation of data. **(B, C)**. %GCV of sample HAI titres before and after normalisation using Pool 1 (sample 17) as a study standard. Samples with a GMT <10 were excluded from analysis (shown as Xs in 5C). The Wilcoxon matched pairs test was used for statistical analysis (H1N1, B Vic and B Yam P<0.0001 ****, H3N2 P=0.0146 *). Data are shown for each virus tested: H1N1 A/Michigan/45/15-like, H3N2 A/HongKong/4801/14-like, B Victoria B/Brisbane/60/08-like and B Yamagata B/Phuket/3073-13-like.

**Supplementary Figure S1B** shows the overall returned HAI data from all laboratories after normalization with Pool 1 (sample 17 in the serum panel). **Figure 3A** shows the GMR of each sample by laboratory before and after normalization, and **Figures 3B, C** shows the %GCV of each sample before and after normalization. GMRs after normalization are closer to 1, particularly for the B strains. Normalization significantly reduces %GCV for all subtypes tested: overall %GCVs are reduced from 68% to 50% (H1N1), from 55% to 51% (H3N2), from 148% to 53% (B Victoria) and from 176% to 94% (B Yamagata).

#### 3.2.5 Impact of using FLUCOP vs. in-house protocols for HAI testing

Six Laboratories carried out testing using both in-house and FLUCOP protocols. Intra-assay agreement (measured by max-min ratios of four coded duplicate samples in the serum panel) was slightly better when testing with the FLUCOP protocol: only 3/256 ratios were >2 with FLUCOP testing compared to 9/256 for in-house testing. Intra-laboratory performance was generally better for FLUCOP testing than in-house testing: 11.5% of data failed to pass the >3.5 max-min ratio requirement when using in-house testing compared to only 6.8% when FLUCOP testing was used (data compared for the 6 laboratories testing both protocols only). These results indicate an overall better performance within a laboratory when using the FLUCOP protocol.

**Figure 4:**
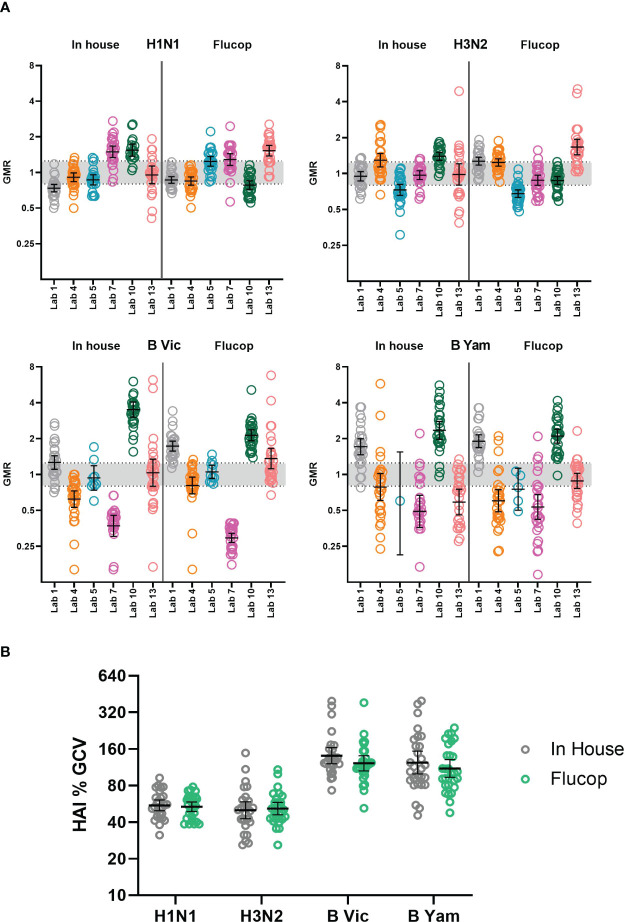
Impact of using the FLUCOP protocol on inter-laboratory variation in HAI testing. **(A)** GMR of each sample HAI titre tested across 6 laboratories using both in-house (left in each graph) and FLUCOP (right in each graph) protocols. GMR was calculated as the ratio of a sample HAI titre compared to the GMT of that sample using in-house methods (for in-house GMRs) or using FLUCOP methods (for FLUCOP GMRs). Geometric mean and 95% CI are plotted in black. The indicative interval of 0.8-1.25 is shaded in grey. Each laboratory has a unique number and colour in all graphical representation of data. **(B)** %GCV for each sample HAI titre tested across 6 laboratories using both in-house protocols (grey) and a FLUCOP protocol (green). Geometric mean and 95% CI are plotted in black.

A comparison of %GCV and GMR for in-house and FLUCOP testing is shown in **Figure 4**. Here the agreement between the 6 sets of in-house data is compared with the agreement between the 6 sets of FLUCOP testing data. GMRs in general do not show substantially closer agreement when using FLUCOP testing compared to in-house testing (**Figure 4A**). There is a marginal trend for GMRs to be closer to 1 using the FLUCOP protocol. %GCVs show a similar result (**Figure 4B**), with slightly lower (but not statistically significant) %GCVs for FLUCOP testing with the B strains, but little or no difference for H1N1 and H3N2 testing.

#### 3.2.6 Impact of using ether split antigen for B virus HAI testing

Laboratories testing B viruses used a mixture of native antigen and ether split antigen in their assays. Separating out laboratories testing with native and split viruses showed that ether split viruses overall gave higher HAI titres, but this does not explain the increased inter-laboratory variability seen for influenza B strains compared to influenza A strains in HAI assays. **Supplementary Figure S2** shows HAI titres for native and ether split antigen for B Victoria (S2A) and B Yamagata (S2B) viruses, with a small but statistically significant increase in HAI titres when using ether split antigen titres. **Figure S2C** shows the %GCV for each sample when tested using native and split antigen. Both groups of native and ether split antigens have high %GCVs all well over 100, and there is no consistent pattern of ether or native antigen having higher or lower %GCVs across the two B lineages; B Yamagata %GCV was significantly lower using ether split antigen and conversely B Victoria %GCV was significantly lower using native antigen. It remains unclear why influenza A viruses gave more consistent results than the influenza B viruses in this study.

### 3.3 MN data analysis

#### 3.3.1 Intra-assay variability

Intra-assay (or within-run) variability was assessed by calculating the maximum to minimum ratio of four pairs of coded duplicates in the serum panel. 92% (334/364) of coded duplicates had maximum to minimum ratios of ≤3.5, with 41% of duplicates being equal. A small number of coded duplicates (5/364) have higher ratios due to conversion of any titres stating >t to 2*t. Labs no.4 and 7 had slightly higher incidence of duplicate ratios >3.5 (33% for Lab no.4 and 25% for Lab no.7 compared to 8.2% across all testing laboratories). Overall, the MN assay is intrinsically more variable within a run than the HAI. Considering the more complex nature of the assay and the use of live cells, this is perhaps not surprising.

#### 3.3.2 Intra-laboratory variability

**Figure 5:**
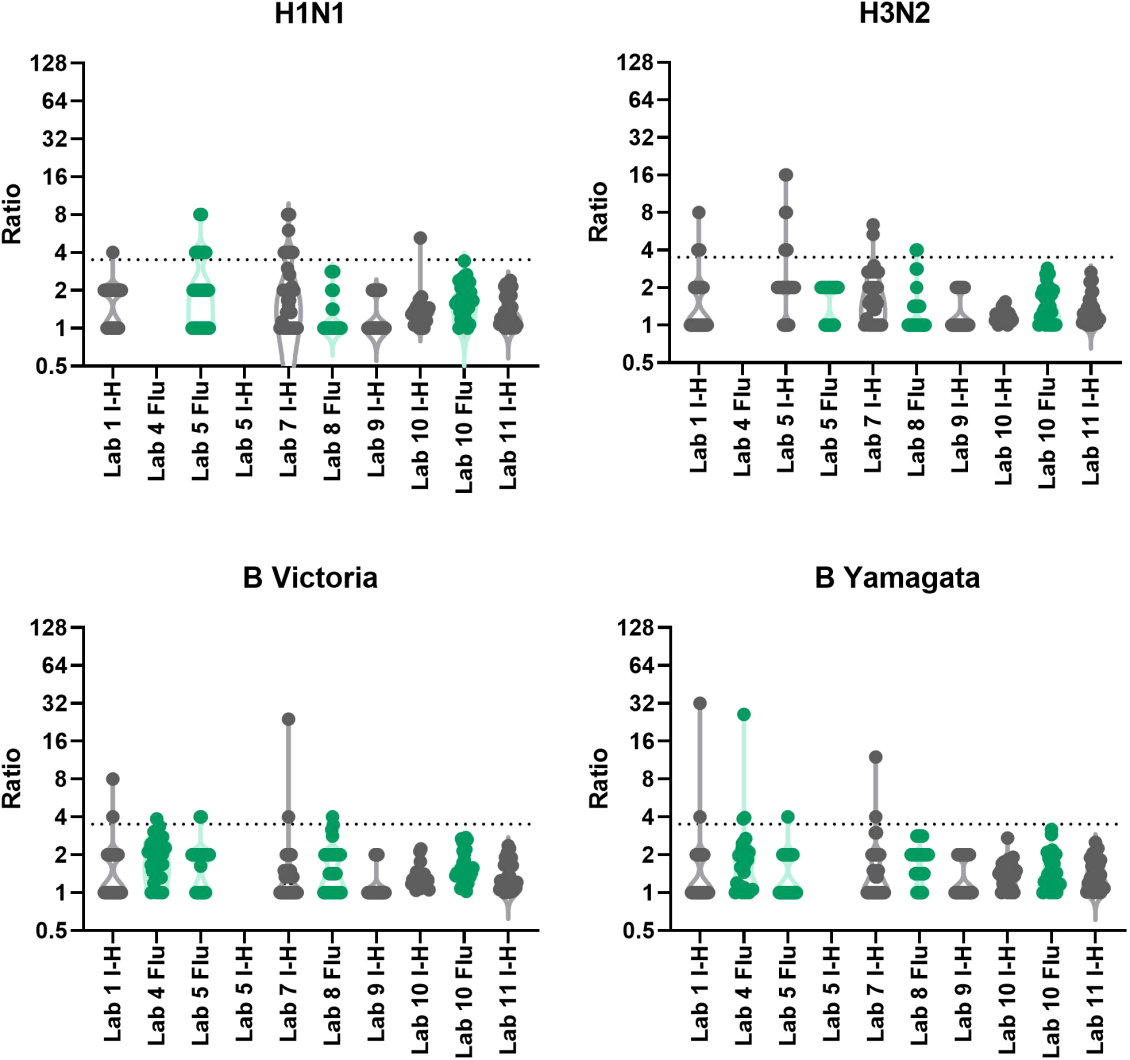
Intra-laboratory variability in MN testing. Maximum to minimum titre ratios for two-three independent MN replicates per laboratory. Dashed line represents the 3.5 cut-off for data exclusion for inter-laboratory variation analysis. Data in grey indicate in-house assays, data in green indicate FLUCOP assays.

We conducted an assessment of intra-laboratory (or between-run) variability by calculating maximum-minimum ratios across the independent replicates performed by the testing laboratories. Several laboratories returned only 2 independent replicates, so we included data where 2 or more independent replicates were carried out. **Table 2** gives the % of samples giving equal, ~2-fold and~4-fold or greater difference in titres across the replicates for each individual laboratory broken down by influenza A subtype and B lineage. The majority (95%) of samples have max-min ratios of ≤3.5, demonstrating good intra-laboratory reproducibility. Unlike the HAI, variability is fairly uniform across laboratories and subtype/lineages with some small laboratory specific differences: Labs no.5 and 7 have higher intra-lab variation for H1N1 testing, and Labs no. 1 and 5 have higher intra-lab variability for H3N2 testing. **Figure 5** plots the max-min ratios for all samples for each testing laboratory, with the cut off of 3.5 as a dotted line. Data above the 3.5 cut off were excluded from further analysis. Overall, 4.8% of the data were excluded (H1N1: 7.9%, H3N2: 5.6%, B Victoria 3.0% and B Yamagata 3.0%).

**Table 2:** MN Intra-laboratory variation - % maximum to minimum ratios across at least 2 replicates.

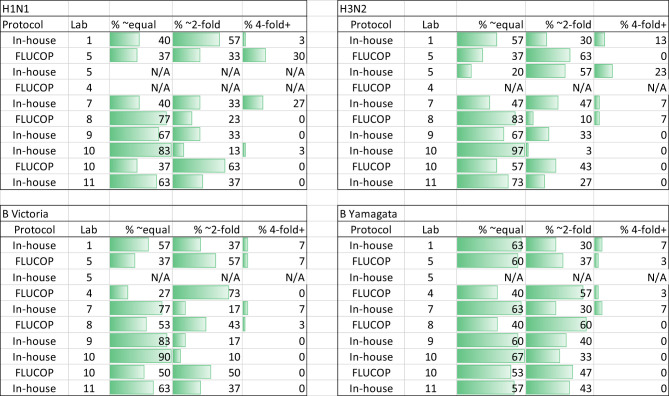

A mixture of discrete and continuous titres were reported. Continuous titres were grouped in the following ranges: equal (≤1.5) ~2-fold (≥1.5 and <4) and 4-fold + (≥4). N/A indicates no data was returned.

#### 3.3.3 Inter-laboratory variability

**Figure 6:**
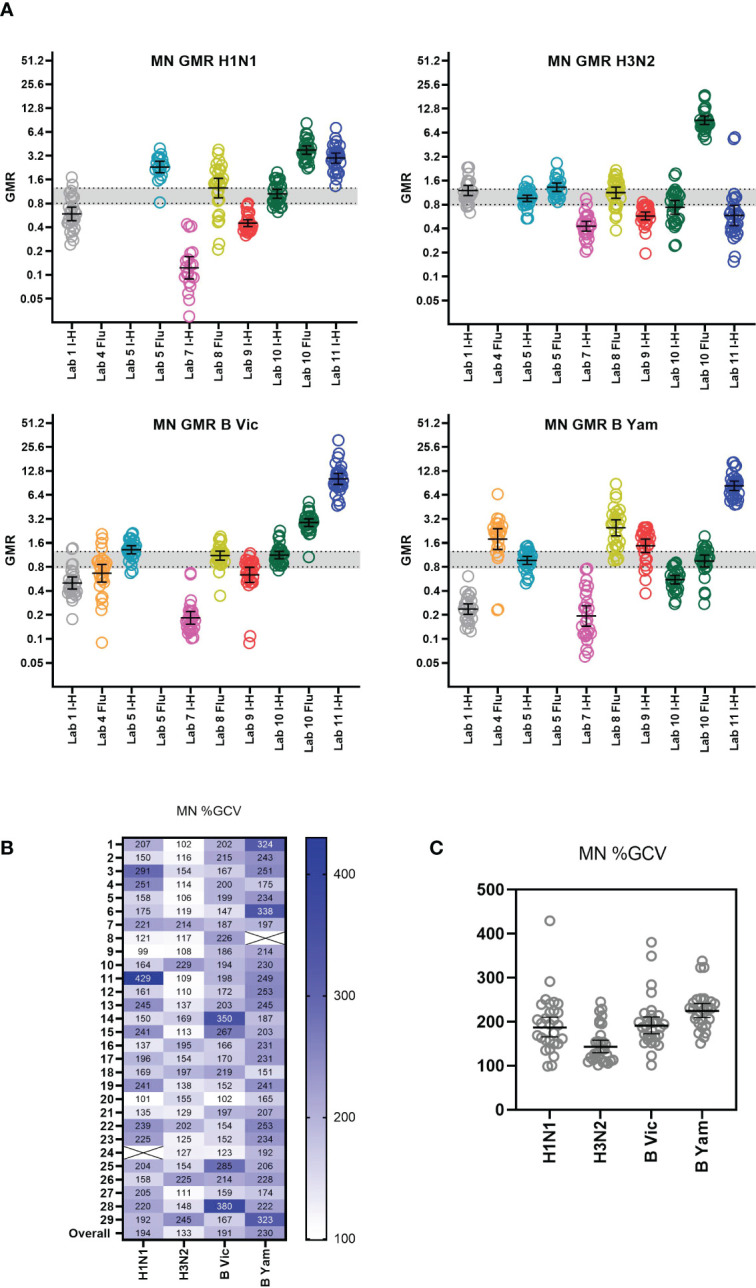
Inter-laboratory variation in MN testing. **(A)** GMR of each sample MN titre compared to the global GMT of that sample across all testing laboratories. Geometric mean of GMRs and 95% CI are shown in black. Laboratories are colour coded and labelled along with protocol type on the x axis. The indicative interval of 0.8-1.25 is shaded in grey. Each laboratory has a unique number and colour in all graphical representation of data. **(B)** %GCV of each sample MN titre across the testing laboratories. Samples with a GMT <10 were excluded from analysis (shown as Xs in the figure). **(C)** %GCV of each sample MN titre plotted by subtype and lineage. Geometric mean of %GCV and 95% CI are shown in as black bars.

**Supplementary Figure S3A** shows the overall returned MN data (after data exclusion as described in statistical analysis) from all laboratories for each influenza A subtype and B lineage. Data from in-house testing are shown in black, data from FLUCOP testing are shown in red. A between laboratory analysis was carried out using two different comparison methods: GMRs of each sample and %GCV of log_10_ titres. GMRs show a large difference in overall titres between different laboratories and protocols (see **Figure 6A**). Unlike HAI data, high levels of variation are seen for each subtype and lineage tested in MN. Ratios in general cluster for each laboratory, suggesting a systematic bias in testing rather than random error. %GCVs across the serum panel were much higher for MN testing than for HAI (see **Figures 6B, C**). Higher inter-laboratory variability is seen for B Yamagata in particular (overall %GCV 230%). %GCV is lowest for the H3N2 subtype (overall %GCV 133%). As only 3 laboratories carried out MN using the WHO ELISA protocol (designated FLU in figures), and not all subtypes/lineages were tested, it is not possible to compare consensus and in-house protocols. Where laboratories tested both side-by-side (Lab no.10 and Lab no.5, **Figure 6A**) titres were generally higher using the WHO protocol.

#### 3.3.4 Impact of a study standard on MN inter-laboratory variation

**Figure 7:**
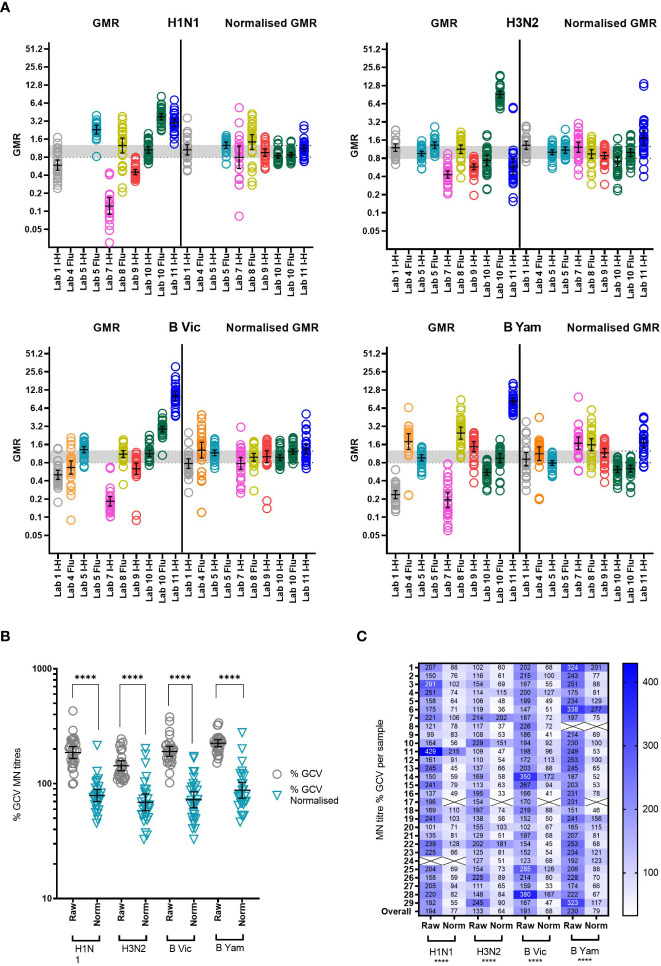
Impact of using a study standard on inter-laboratory variation in MN testing. **(A)** GMR of the MN titre of each sample before and after normalisation using Pool 1 (sample 17) as a study standard. Negative samples (MN GMT <10) were excluded from analysis. Where a laboratory did not return a valid titre for the study standard in each reported run, that laboratory was excluded from analysis. Geometric mean and 95% CI are shown in black. The indicative interval of 0.8-1.25 is shaded in grey. Each laboratory has a unique number and colour in all graphical representation of data. **(B, C)** %GCV of each sample MN titre before (raw) and after (norm) normalisation using Pool 1 (sample 17) as a study standard. The Wilcoxon matched pairs test was used for statistical analysis (in each case P<0.0001****) Data are shown for each virus tested: H1N1 A/Michigan/45/15-like, H3N2 A/HongKong/4801/14-like, B Victoria B/Brisbane/60/08-like and B Yamagata B/Phuket/3073-13-like. Negative samples (MN GMT <10) were excluded from analysis (shown as Xs in **C**).

We included two pools of human sera as study standards within the serum panel tested by participating laboratories. The impact of normalization using these study standards was explored by comparison of %GCV of log_10_ titres before and after normalization with one of these pools (sample 17) and a comparison of GMRs before and after normalization. **Supplementary Figure S3B** gives a summary of normalized MN titres across the 8 testing laboratories. It is clear that after normalization titres cluster closer together for each sample. **Figure 7A** shows the GMRs of each sample by laboratory before and after normalization, with GMRs clearly closer to 1 after normalization. **Figures 7B, C** shows the %GCV of each sample before and after normalization. Post normalization, %GCV are much improved. A statistically significant decrease in %GCV of over 50% was observed for all four subtypes/lineages tested: overall %GCV was reduced from 194% to 77% (H1N1), from 133% to 64% (H3N2), from 191% to 68% (B Vic) and from 230% to 79% (B Yamagata).

## 4 Discussion

The aim of this EQA study was to assess the feasibility of carrying out an EQA scheme for seasonal influenza serology testing, and to provide participating laboratories with a valuable data set giving evidence of a laboratory’s performance within a peer group of laboratories routinely using influenza serology assays. Ideally an EQA scheme would use commutable materials as test samples that have been given assigned values through testing with a reference measurement procedure, with a good understanding of uncertainty within the measurement (5). This is not possible for seasonal influenza testing, where the complex immunological exposure of human donors and the lack of reference measurement procedures for biological assays make such assigned values impossible. As an alternative, we have used the peer group of laboratories in this study to generate global geometric mean titres (GMTs) and used these as the ‘assigned values’ for comparison (i.e. comparison of each laboratory to the mean of all laboratories). This method has the obvious disadvantage that low numbers of participating laboratories and high levels of variation can have a significant impact on the uncertainty of GMTs as assigned values (5), making it harder to set limits on acceptance criteria for (or draw conclusions from) the data. In this study, we had 16 laboratories returning data – a number that was likely impacted by the ongoing Covid-19 pandemic. This represents a limitation of the study, however, the dataset collected is still valuable and serves to highlight where improvements in intra-assay, intra-laboratory and inter-laboratory agreement can be made.

We have not sought to implement limits to which we consider inter-laboratory performance to be acceptable or not. Instead, we have set arbitrary ranges that laboratories may consider (for example the indicative range of 0.8-1.25 for geometric mean ratios) and have put other measures of inter-laboratory variation into the context of existing literature for comparison (for example the %GCVs of samples across the testing laboratories). Intra-assay performance and intra-laboratory performance have been analyzed and described as valid or failed based on standard and published criteria for serology assays (6). For these analyses, laboratories can see whether improvements are required to reduce intra-assay (within-run) and intra-laboratory (between-run) variability. In this study the most commonly observed incidence of variability for HAI testing appears to stem from the balancing of viruses from run to run, with several laboratories having much higher titres in one run compared to the next. Care should be taken to balance viruses to 4 HAU/25ul (or equivalent) at the start of each assay run and ensure adequate training is given to reduce differences in operators/technicians, although it is possible that other factors (such as batch to batch variation in turkey red blood cells and reading of HAI plates) may impact upon this.

This study highlights that variability in HAI testing is significantly higher for influenza B strains compared to influenza A strains. B Victoria and particularly B Yamagata lineage viruses had high %GCV with both native and ether split antigen, however our data suggests that ether splitting the B Yamagata lineage virus reduces inter-laboratory variability. This is in line with a previous study demonstrating that ether split B Yamagata had lower %GCV than native antigen using in-house protocols (2), although interestingly this was not the case for a B Victoria antigen. Why high %GCVs are frequently observed for B Yamagata lineage viruses, and why the use of ether splitting appears more beneficial for B Yamagata compared to B Victoria lineage viruses is not clear, perhaps the quality and stability of ether split antigens may differ between B strains/lineages.

High levels of inter-laboratory variability have been observed for B lineage viruses in a previous FLUCOP study (2) with %GCVs of 89% for B Victoria and 117% for B Yamagata (in-house testing with in-house antigen) compared to 50% for H1N1 and 70% for H3N2 strains. In this study %GCVs for B lineage viruses were more than double compared to %GCVs for influenza A viruses. Participants of this EQA reported that despite careful balancing of B lineage viruses during HAIs, run-run differences in HAI titres were high. It is possible that batch-batch variations in TRBCs have a greater impact on B viruses due to differences in receptor binding affinities between influenza A and B strains. B viruses are known to undergo egg adaptation, augmenting binding to avian sialic acid residues (7), however there is evidence that egg adaptation in B viruses contrasts to that seen in influenza A viruses, in that adaptation may involve multiple viral factors resulting in an increased ability to bind α2,3 receptors in eggs without losing the avidity for human receptors (8). Studies additionally indicate that the steric configuration of asialyl sugars of the receptor analogue may have a greater impact on binding avidity of B influenza viruses than the analogue being an α2,3 or α2,6 linked sialyl-glycan (8, 9). It is possible that subtle differences in asialyl sugar configuration between TRBC batches, and/or mutations during egg adaptation may have a greater impact on influenza B virus HA binding compared to influenza A viruses, although this remains to be seen. Regardless of the cause, this run-run variation was dramatically reduced when results were normalised with a standard, supporting the development of seasonal influenza standards.

The majority of published figures on inter-laboratory %GCV focus on influenza A viruses. The overall HAI %GCVs observed in this study for H1N1 (69%) and H3N2 (57%) are in line with or lower than previous studies (10–14).

For HAI, the use of a consensus protocol alone (without standardized reagents) did not show a statistically significant difference for the 6 laboratories that tested FLUCOP and in-house protocols side by side. Although this study was not initially designed to compare FLUCOP and in-house methods, our analysis shows agreement with previous studies that demonstrate a stricker level of harmonization than protocol sharing is required to be effective in reducing inter-laboratory variability (2, 15).

It was clear that the use of a pool of serum as a study standard was effective in reducing inter-laboratory variation, again consistent with published studies (2, 10, 13, 15). This adds to the growing body of evidence in favor of developing seasonal influenza serology standards.

MN assays had intrinsically higher levels of variability within a run (reflected by 8.2% of coded duplicates failing in MN testing compared to 1.8% for HAI) but run to run variation was lower (4.8% of data are excluded from MN analysis due to high between-run variability compared to 11% of data for HAI). Much of the excluded data for HAI comes from strain specific difficulties in balancing viruses between runs (particularly for B strains), skewing the data slightly. The considerably higher levels of inter-laboratory %GCV seen for MN assays (range of 133%-230%) likely reflect the higher diversity in protocols for MN assays, with multiple readout methods. Other studies agree that MN assays have higher inter-laboratory variation than HAI (10, 13, 14). In comparison to previous studies, the %GCV observed for absolute titres in this EQA is consistent, with values in the hundreds – in fact %GCVs for MN assays have previously been reported with higher values than seen here: H3N2%GCVs in the range of 256-359 (14), H1N1%GCVs in the range of 204-383 (13) and H5N1%GCVs in the range of 112-185 (10). In each of these studies, as seen here, the use of a serum standard significantly reduced inter-laboratory variation. Our data showed a reduction in %GCV of more than half, to less than 80% for each influenza A subtype and B lineage tested after normalization.

This paper represents a feasibility study for carrying out a regular EQA for seasonal influenza serology, gauging the interest of laboratories for participating in such an activity. Some further considerations should be taken into account for such an EQA scheme in the future. As in-house stocks of antigen were tested in this EQA study, it is possible that viruses will have acquired changes during propagation in eggs that will vary from testing lab to testing lab. We also allowed testing of wild type or reassortant antigens that are antigenically identical, though it is known that antigen type (WT, egg or cell passaged) can have an impact on HAI titre and inter-laboratory variation (manuscript in preparation). Perhaps sequencing of HA/NA genes of tested antigen would provide useful information on any variability in in-house antigen stocks. Additionally, it would be interesting to carry out a comparison of in-house protocols used within such a study. HAI protocols have been shown within the FLUCOP consortium to vary at every stage of the assay (2), and although outside the scope of this feasibility study, protocol comparison may reveal important differences and present opportunities for harmonization of serology testing.

We received positive feedback from multiple participants of this feasibility study, demonstrating the positive impact such a scheme would have on identifying laboratory specific issues with serology testing, raising awareness of harmonized protocols for serology testing and raising awareness of the value of biological standards in reducing inter-laboratory variation.

## Data availability statement

The raw data supporting the conclusions of this article will be made available by the authors, without undue reservation.

## Ethics statement

The studies involving human participants were reviewed and approved by Universitair Zeikenhuis Gent, Commissie vor medische ethiek (committee for medical ethics) Belgian registration number B670201733136. The patients/participants provided their written informed consent to participate in this study.

## FLUCOP consortium collaborators

Marie-Clotilde Bernard, Barbara Camilloni, Maria Rita Castrucci, Marco Cavaleri, Annalisa Ciabattini, Frederic Clement, Simon De Lusignan, Oliver Dibben, Susanna Maria Roberta Esposito, Marzia Facchini, Felipa Ferreira, Sophie Germain, Sarah Gilbert, Stefan Jungbluth, Marion Koopmans, Teresa Lambe, Geert Leroux-Roels, Donata Medaglini, Manuela Mura, Nedzad Music, Martina Ochs, Thierry Ollinger, Albert Osterhaus, Anke Pagnon, Giuseppe Palladino, Elena Pettini, Ed Remarque, Leslie Reperant, Hanna Sediri Schön, Sarah Tete, Alexandre Templier, Serge van de Witte, Gwenn Waerlop, Ralf Wagner, Brenda Westerhuis, Fan Zhou.

## Author contributions

Conceptualization and study design JW, OE, CC. Laboratory work JW, SM, CT, SR, LC, SS-O, AF, SJ, ML, WW, SL, RC, LM, AB, CF, JD, JE, IM, J-SM, MA, AS, NK, SH, EMa. Data analysis JW. Writing and Editing JW, OE, CT, SM, EMo, AF, SJ, ML, WW, CW, RC, LM, AB, RW, CC, SW, SH, KH, BL, DD. All authors had full access to the data and approved the final draft of the manuscript before it was submitted by the corresponding author.
