# Supplementary material for: An external quality assessment feasibility study; cross laboratory comparison of haemagglutination inhibition assay and microneutralization assay performance for seasonal influenza serology testing: A FLUCOP study
Source: Front Immunol. 2023 Feb 28;14:1129765. doi: 10.3389/fimmu.2023.1129765 (PMC10011125; doi:10.3389/fimmu.2023.1129765)
Supplement: Supplementary file 1 [file DataSheet_1.docx]

Supplementary Material

An External Quality Assessment feasibility study; cross laboratory comparison of haemagglutination inhibition assay and microneutralisation assay performance for seasonal influenza serology testing: a FLUCOP study.

Joanna Waldock^*^, Carol Weiss, Wei Wang, Min Z Levine, Stacie N Jefferson, Sammy Ho_,_ Katja Hoschler, Brandon Z Londt, Elisa Masat, Louise Carolan, Stephany Sánchez-Ovando , Annette Fox , Shinji Watanabe, Miki Akimoto, Aya Sato, Noriko Kishida, Amelia Buys, Lorens Maake, Cardia Fourie, Catherine Caillet, Sandrine Raynaud, Richard J Webby, Jennifer DeBeauchamp, Rebecca J Cox, Sarah L Lartey, Claudia M Trombetta, Serena Marchi, Emanuele Montomoli Iván Sanz Muñoz, José María Eiros, Javier Sánchez-Martínez, Danny Duijsings, FLUCOP consortium collaborators, Othmar G Engelhardt.

***Corresponding author email address and address**

[Joanna.waldock@nibsc.org](mailto:Joanna.waldock@nibsc.org)

# Supplementary Figures and Tables

## Supplementary Figures


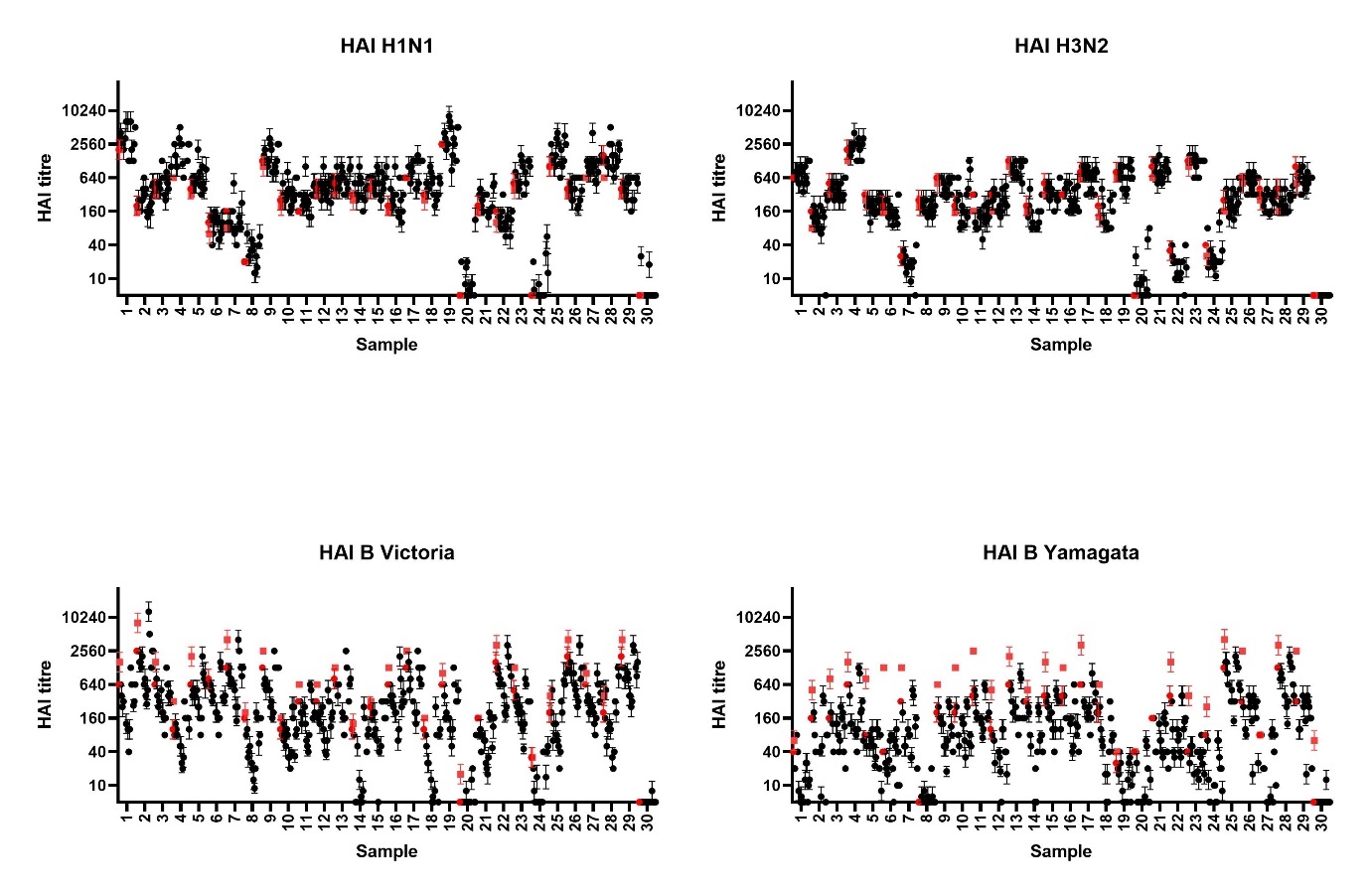
**S1A**

**S1B**

**Supplementary Figure S1. Summary of returned HAI data from participating laboratories [S1A] before and [S1B] after normalisation using a study standard**. The GMT of each sample per laboratory is plotted (with geometric standard deviation as error bars). Data was excluded where less than 2-3 independent replicates were returned, or where replicates had a min-max ratio >3.5. A per-run normalisation was carried out using Pool 1 (sample 17) as a study standard. Data is shown for each virus tested: H1N1 A/Michigan/45/15-like, H3N2 A/HongKong/4801/14-like, B Victoria B/Brisbane/60/08-like and B Yamagata B/Phuket/3073-13-like. Data from in-house testing is shown in black, data from FLUCOP testing is shown in red.

**Supplementary Figure S2. Impact of ether splitting on HAI titre and inter-laboratory variation**. B Victoria (**S2A**) and B Yamagata (**S2B**) HAI titres of the sample panel when testing using native (grey, circles) antigen (4 laboratories returning 6 data sets) or ether split (blue, squares) antigen (6 laboratories retuning 9 data sets). Geometric mean and 95% CI are shown in black. **S2C**. %GCV of each sample across all laboratories testing with native antigen (Native, grey, circles) or ether split antigen (+ Ether, blue, squares). The geometric means and 95%CI are shown in black. Samples with a GMT <10 were excluded from analysis. Native and Ether split titres were compared using the Mann-Whitney U Test ***P=0.0002, *P=0.0296.

**S3A**

**S3B**

**Supplementary Figure S3. Summary of returned MN data [S3A] before and [S3B] after normalisation using a study standard**. The GMT of each sample is plotted (with geometric standard deviation as error bars). Data were excluded where less than 2-3 independent replicates were returned, or where replicates had a min-max ratio >3.5. A per-run normalisation was carried out using Pool 1 (sample 17) as a study standard. Data are shown for each virus tested: H1N1 A/Michigan/45/15-like, H3N2 A/HongKong/4801/14-like, B Victoria B/Brisbane/60/08-like and B Yamagata B/Phuket/3073-13-like. Data from in-house testing are in black, data from FLUCOP testing in red.
